# Hollow core optical fiber enabled by epsilon-near-zero material

**DOI:** 10.1515/nanoph-2024-0025

**Published:** 2024-03-06

**Authors:** Leon Zhang, Stuart Love, Aleksei Anopchenko, Ho Wai Howard Lee

**Affiliations:** Department of Physics & Astronomy, 531098 University of California, Irvine, CA 92697, USA; 531098 Beckman Laser Institute and Medical Clinic, University of California, Irvine, CA 92697, USA

**Keywords:** epsilon-near-zero material, hollow core optical fiber, optical fiber, zero-index material, fiber laser

## Abstract

Hollow core optical fibers of numerous guiding mechanisms have been studied in the past decades for their advantages on guiding light in air core. This work demonstrates a new hollow core optical fiber based on a different guiding mechanism, which confines light with a cladding made of epsilon-near-zero (ENZ) material through total internal reflection. We show that the addition of a layer of ENZ material coating (e.g. indium tin oxide layer) significantly reduces the loss of the waveguide compared to the structure without the ENZ layer. We also show that the propagation loss of the ENZ hollow core fiber can be further improved by integrating ENZ materials with lower loss. This study presents a novel type of hollow core fiber, and can find advanced in-fiber photonic applications such as laser surgery/spectroscopy, novel gas-filled/discharge laser, in-fiber molecular/gas sensing, and low-latency optical fiber communication.

## 1 Introduction

Solid core silica optical fibers have been studied extensively in the past few decades and used in a broad range of important applications, including telecommunication, remote sensing [1], [2], imaging [3], spectroscopy [4], etc. Light is confined within the fiber due to total internal reflection, a mechanism which prevents light from refracting outside of the core under these conditions. This type of fiber, though robust, transmits energy and information in the glass at a speed far from the maximum possible speed in vacuum/air, since the speed of transmission is dependent on the core’s refractive index which is around 1.5 in telecommunication wavelengths. Furthermore, conventional glass core fibers have a limited maximum power, partially because glass can be damaged at sufficiently high power [5], [6], and glass still exhibits optical nonlinearity, changing the optical properties of the media thus leading to distortion of information [7].

Instead of guiding light in glass media, researchers developed hollow core optical fibers to improve upon the speed of transmission and remove undesirable nonlinear effects [8], [9], [10]. With the light guiding in the air-core, the speed of light is close to that in vacuum, and the nonlinear effects and damage threshold of air of much less consideration compared to traditional fiber material such as germanium-doped silica glass.

One of the main applications for these hollow core fibers is in high-sensitivity chemical/gas sensing. Indeed, because the medium of light transport is air, the electromagnetic radiation confined is highly sensitive to the changing index of the air composition. It has been shown to accurately measure the spectra of acetylene gas [11], methane [12], [13], and carbon monoxide [12]. The hollow channels in hollow core fiber allow infiltration of chemical samples, leading fiber based optofluidic and photocatalytic devices for photochemistry and particles sensing/trapping applications [14], [15], [16], [17]. Due to the higher transmission speed of light in air-core optical fibers, they have been used for low latency and high power optical communications [18], [19].

Different mechanisms to realize hollow core fibers have been reported in recent years, such as the photonic bandgap [20], [21], [22], [23], [24], [25], Kagome-type hollow core guidance [26], [27], [28], anti-resonant guidance [9], [29], [30], [31], [32], omnidirectional reflection [33]. However, these designs typically involve complicated fiber geometry and can only allow for transmission in narrow spectral ranges. In addition, these hollow-core fibers remain fundamentally limited by the existing cladding glass materials and suffer from scattering loss due to the holey cladding structures.

In this letter, we demonstrate a fundamentally new air-guided optical fiber that uses newly developed “zero-refractive index” material as a guiding medium. In this transformative optical guiding concept, light will be guided in the “air” surrounded by the zero-refractive index materials via total internal reflection at the maximum speed of light with extremely high-power transport, overcoming the significant limitations of conventional optical fiber where light properties are largely limited by the glass core material. This new type of hollow core optical fiber could be important for advanced in-fiber laser applications such as laser surgery/spectroscopy, novel gas-filled/discharge laser, in-fiber molecular/gas sensing, and low-latency optical fiber communication. And because of the simple geometry of the platform, it could eliminate the need to design such complicated shapes to maintain core mode within the air core, improving fabrication techniques.

Epsilon-near-zero (ENZ) materials, or zero refractive index materials, which have its real part of the relative permittivity, Re(*ε*), reduced to zero at a certain wavelength, have been studied for its unique optical properties and multiple emerging applications in photonics [34], [35], [36] including enhanced optical nonlinearity [37], [38], [39], [40], extreme optical confinement [41], enhanced quantum emission and directive emission [42], [43], [44], and strong light absorption [45], [46], [47]. While ENZ optics show the promise for extreme light manipulation, most of the studies of ENZ materials have been restricted on optical thin films and planar structures, limiting the development of novel ENZ optical devices and applications. To extend the ENZ optics to different optical platform, we have previously integrated ENZ materials into D-shaped single mode optical fibers and nano-bore optical fibers to efficiently excite the ENZ mode. We showed that highly confined ENZ optical mode can be excited in thin ENZ layer coated on optical fibers, leading to efficient electrical/optical switching and optical sensing [48], [49]. Instead of using thin ENZ thin film for mode excitation, in this work, we investigate an air-guided hollow core optical fiber based on thick ENZ coating of hollow core channel.

## 2 Results and discussions

**Figure 1:**
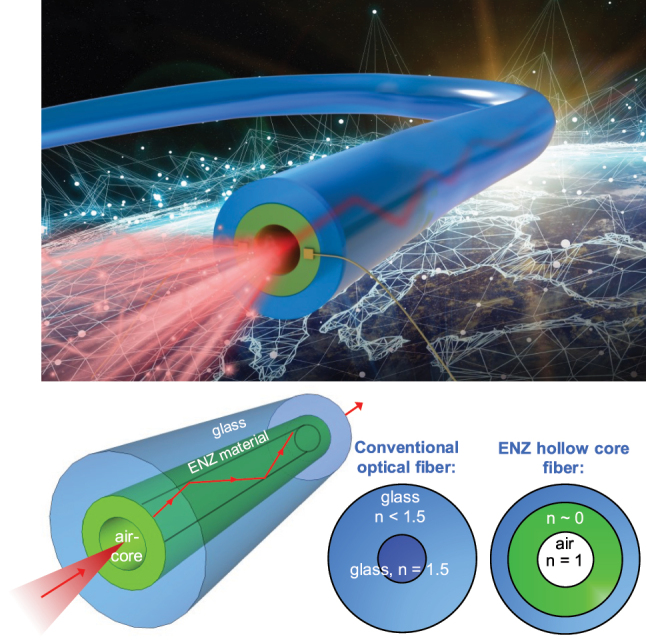
Schematic of the novel hollow core ENZ fiber. Near the ENZ wavelength, the ENZ material has low index of refraction which is less than that of air, leading to total internal reflection condition. The diameter of the core as well as the thickness of the ENZ layer can be varied and result in different optical properties.

As shown in Figure 1, the hollow air core where light propagates through with a cladding made of ENZ material. Near the ENZ wavelength, the material has an index of refraction less than that of air. Thus, this structure creates an index profile to that of a conventional step-index fiber, with a high index core, and a lower index cladding, which allows for total internal reflection.

**Figure 2:**
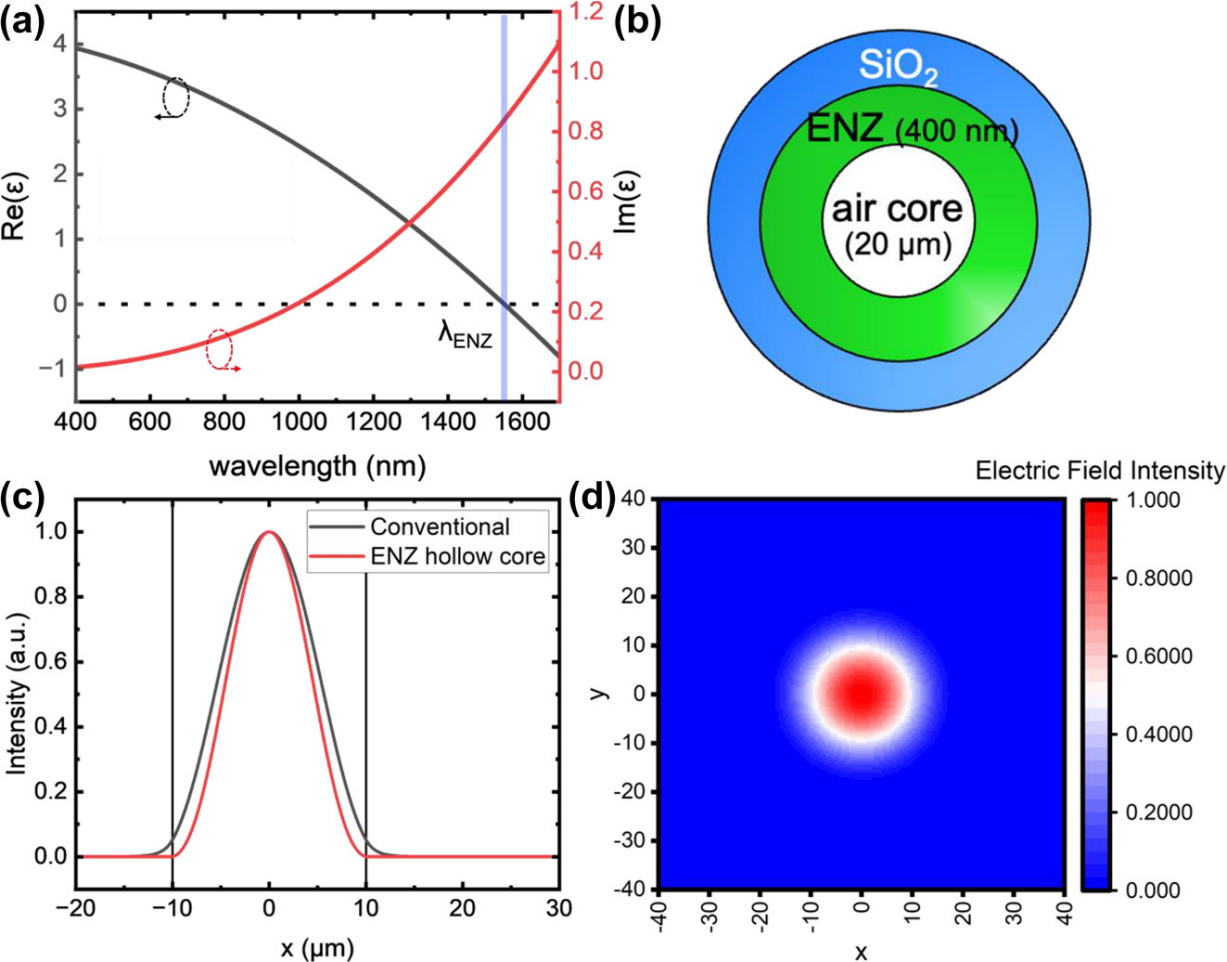
Simulated mode profile of ITO ENZ material coated hollow core optical fiber. (a) Complex permittivity of the ITO ENZ material. The ENZ wavelength is 1550 nm. (b) Schematic of the ENZ fiber. (c) Comparison between fundamental mode electric field distribution for a conventional optical fiber and an ENZ hollow core fiber, both calculated with a core size of 20 µm as indicated by the black lines, the ENZ thickness is 400 nm (d) fundamental mode electric field relative intensity distribution of the ENZ hollow core fiber with core diameter of 20 µm and ENZ thickness of 400 nm.

The air-core’s guiding properties of the ENZ fiber are modelled with full-wave electromagnetic simulations (*Lumerical Inc*.). To model the ENZ layer, a ring with inner diameter *d*_*core*_ and outer diameter *d*_*core*_ + *d*_*ENZ*_ was used. It was chosen this way to keep the air core diameter of the fiber constant, since studies have shown that the loss of a hollow capillary fiber is related to the core diameter with 1/*R* [3] dependence [50]. Indium-tin-oxide (ITO) is used as the ENZ medium in the analysis. The frequency dependent complex permittivity of ITO was calculated using the Drude model:

$$\varepsilon \left(\omega \right)=1-\frac{\omega_{p}^{2}}{\omega^{2}+i\Gamma \omega }$$

where Γ is the inverse of the average time between collisions of free charges and *ω*_*p*_ is the plasma frequency, which is defined as:

$$\omega_{p}=\frac{N_{e}q^{2}}{\varepsilon_{0}m_{e}}$$

where *N*_*e*_ is the free carrier density, *q* is the charge of the electron, *ε*_0_ is the permittivity of free space and *m*_*e*_ is the effective electron mass. By changing the carrier density of the material, the material can have different crossing points where the real part of the permittivity crosses zero which can be controlled when depositing materials (e.g. atomic layer deposition, sputtering, etc.). The parameters used in the model came from experimentally measured permittivity from an ITO sample fabricated by sputtering technique with an ENZ wavelength of ∼1550 nm (Figure 2(b)) (see ITO material and optical properties in Methods and Supplementary Material).

The electric field relative intensity distribution is similar between the conventional optical fiber and ENZ hollow core fiber, with the latter having a slightly more concentrated distribution within the air core. We show the field confinement of the hollow core fiber in comparison with the 400 nm ENZ coated fiber in Figure 2(c). As the figure shows, the ENZ layer creates a stronger field confinement within the core with respect to the uncoated fiber. In the thinner films (<400 nm), there is also additional coupling to the ENZ film (see Supplementary Material). The fundamental mode of the ENZ coated hollow core fiber at the ENZ wavelength of 1550 nm for ENZ layer thickness of 400 nm is depicted in field profile in Figure 2(d). The fundamental core mode has a Gaussian-like electric field distribution (HE_01_ mode), where most of the field intensity is confined within the central air core.

**Figure 3:**
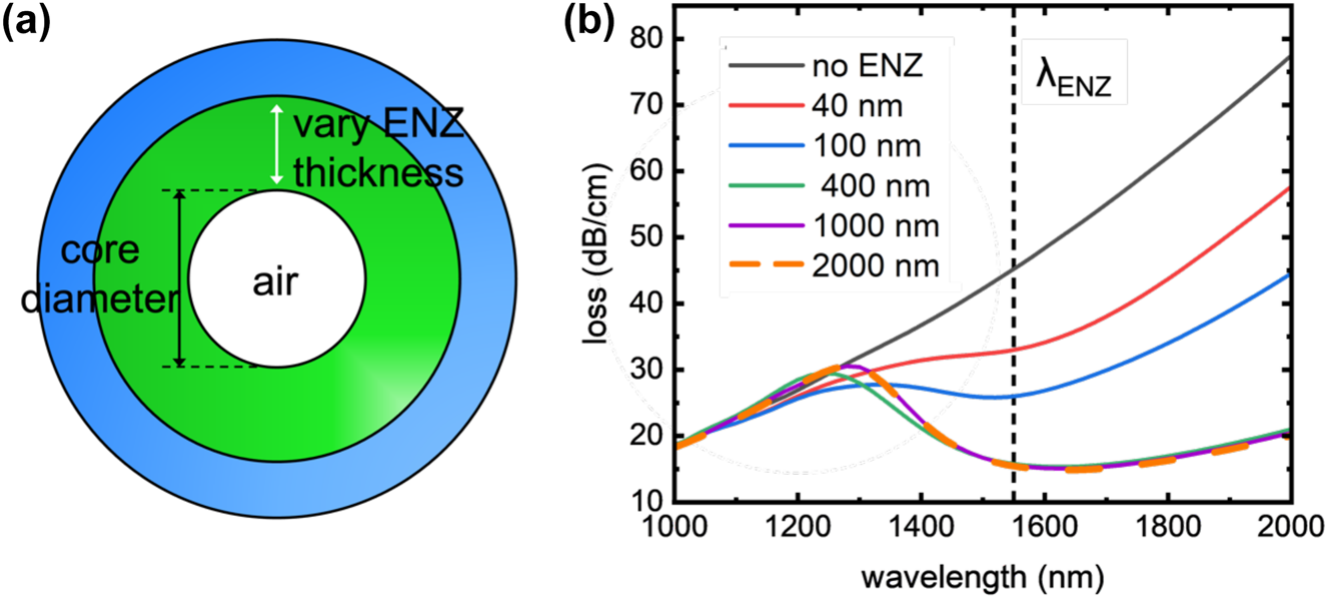
Propagation loss for fundamental mode of ENZ hollow core optical fiber with varying ITO coating thicknesses. (a) Study of propagation loss of ENZ optical fiber by varying ENZ layer thickness. The fiber has a fixed 20 µm air core diameter for all ENZ thickness. (b) Simulated transmission loss of the hollow core fiber for different ENZ thicknesses. The ENZ wavelength is 1550 nm.

We further investigate the transmission properties of the fundamental mode of ENZ hollow core fiber for different fiber geometries. A frequency dependent effective index of the fundamental mode was calculated and the modal loss curves were calculated in the same way ( $loss=-20log_{10}e^{-2\pi k/\lambda_{0}}$ , where *k* is the imaginary part of the effective index). As shown in Figure 3, when fixing the fiber air core diameter at 20 µm and varying the thickness of the ENZ wall, the fiber loss spectrum changes drastically. When there is no ENZ material present and the fiber simply consists of a hollow core with glass cladding, the transmission loss increases linearly with increasing wavelength (Figure 3(b)). At the lowest losses in the above configuration, we have 14.9 dB/cm in losses, with a FWHM of approximately 450 nm near the ENZ wavelength of 1550 nm.

**Figure 4:**
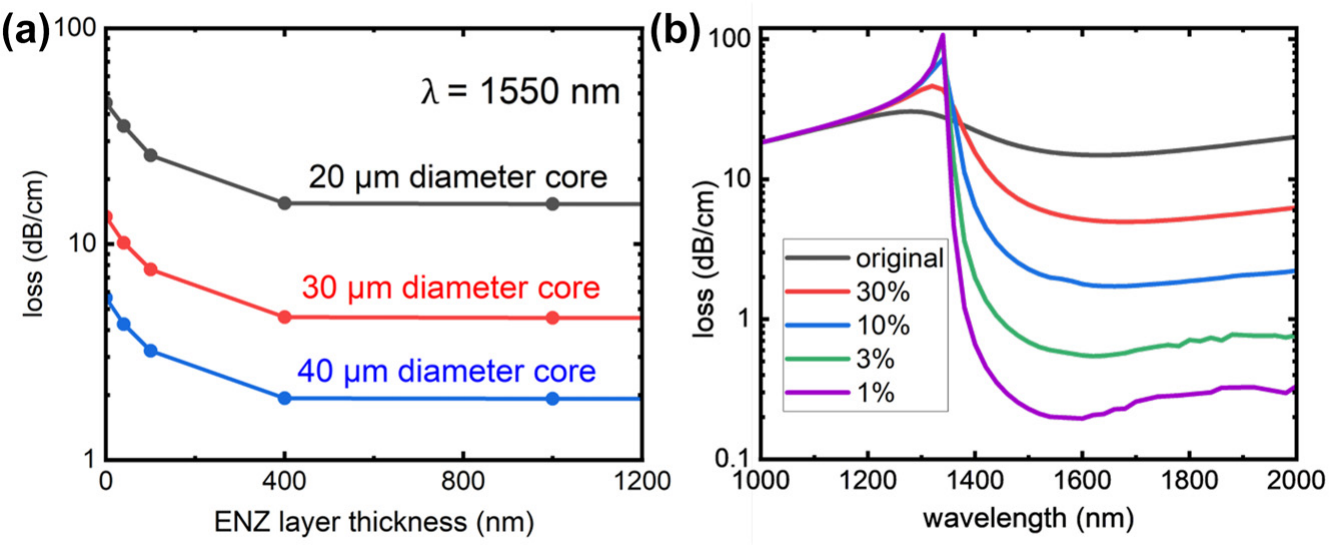
Propagation loss for fundamental mode of ENZ hollow core optical fiber with varying core diameters and ITO losses. (a) Simulated loss spectra for hollow core ENZ optical fibers with different core diameters (20 μm, 30 μm, 40 μm) and ENZ layer thicknesses at the ENZ wavelength of 1550 nm. (b) Simulated modal loss of the fundamental mode of the ENZ hollow core fiber with different ENZ material’s optical losses. Different materials which have their imaginary part of the permittivity reduced to some percentage of the original value of the ITO were modelled. The core diameter is fixed at 20 μm and ENZ thickness set arbitrarily large.

An additional parameter that can influence the performance of the fiber is the diameter of the air core. To investigate if the previously found behavior with varying ENZ thickness has changed, we simulated the fiber’s loss at 1550 nm with 3 different core diameters, while changing the ENZ layer’s thickness. As shown in Figure 4(a), regardless of the fiber core diameter, increasing the thickness of the ENZ layer still results in the same decreasing transmission loss, up to a certain amount. The only variance is that there is an overall decrease in loss for larger air core diameter, which is consistent with the behaviors of other hollow core fibers [50]. Thus, the mechanism behind the design is unaffected by the changing diameter, and the dimensions of the fiber can be easily modified to better suit the need of an application.

Next, we investigate the propagation loss of the air-guided ENZ optical fiber related to the loss of the ENZ materials (imaginary part of the permittivity, Im(*ϵ*)). To simulate the behavior of an ENZ material with lower loss, different materials which have their imaginary part of the permittivity reduced to some percentage of the original value of the ITO were modelled (see Supplementary Material S1 for details). As shown in Figure 4(a), the modal loss at the ENZ wavelength converge to a minimal value at higher ENZ wall thickness, thus, to minimize the modal loss around the ENZ wavelength, the structure was simulated with an arbitrarily large ENZ wall thickness. The result, as depicted in Figure 4(b), shows that the modal loss of the ENZ fiber at the ENZ wavelength is reduced by almost an order of magnitude for each order of magnitude reduction in material loss, demonstrating the potential of low loss air guiding in ENZ optical fiber. Note that a high loss peak is observed at ∼1300 nm, which we attribute to an enhanced transmittance of the ENZ layer on silica and leakage of the guided mode within the capillary (see Supplementary Material S2).

Though the results presented were simulated for a structure using indium tin oxide (ITO) ENZ material, the design is not limited to any particular choice of ENZ material. Many types of ENZ material have been realized [34], such as metals, doped semiconductors, phononic materials, and more. Depending on considerations such as the operation wavelength range, material availability, and ease of fabrication, one can realize the fiber structure and utilize the same principle with a more suitable material. It is noteworthy that advancements have been made in developing ENZ materials with significantly lower optical losses. Dysprosium-doped cadmium oxide (CdO:Dy) thin films exhibit NIR-mid IR losses of 0.13–0.16 [51] while aluminum nitride can achieve an imaginary permittivity of 0.02 at its ENZ wavelength [52] The proposed design further presents the potential for optimization with ZnO:Al/ZnO multilayers offering an out-of-plane imaginary permittivity as low as 0.003 at 1885 nm [53] and CdO:Dy multilayers demonstrating out-of-plane losses of 0.065 [54].

Though metal coated hollow core cylindrical waveguides have been studied [55], ENZ materials based on doped semiconductors can exhibit significantly lower materials loss (Im(*ϵ*) < 0.3) at an ENZ wavelength in the near infrared region [56], making it ideal for light guidance and other photonic applications required air-core. In addition, metallic coatings have exceptional losses in the visible regime, making them unusable for applications needing that section of the optical spectrum. Furthermore, as the field around ENZ material continuously evolves, other ENZ materials with lower losses are being developed. It is also important to note that the conducting oxide ENZ materials generally exhibit high damage threshold [51], [57]. Metal nitride ENZ materials such as TiN, can withstand a high laser intensity without damage owing to their properties as a refractory metal. For example, a damage threshold of few TW/cm^2^ could be achieved in TiN [58], [59] showing its suitability for use in high power laser hollow core guiding applications.

Finally, the simple geometry of the ENZ hollow core fibers could be fabricated directly through atomic layer deposition, which has been shown to be able to deposit ENZ material inside the hollow channels of the fiber through a conformal layer by layer process [48], [60]. Thus, the desired structure can be achieved by depositing ENZ material on commercially available hollow core glass capillaries. For fabricating long ENZ hollow core fibers in hundreds of meters to kilometers, one can coat the cane with ENZ material via deposition or wet chemistry techniques, and draw the fiber directly with fiber drawing technique, however, it is beyond the scope of this paper.

## 3 Conclusions

In this work, we demonstrate that ENZ material can be used as the cladding to guide light in an air-core optical fiber. We show that the ENZ waveguide structure supports a fundamental mode where light is strongly confined in an air core, and the inclusion of ENZ materials enhances light guidance through a glass capillary and reduces the propagation loss. Furthermore, the fiber can be further improved through the development of ENZ materials with lower loss and with different fiber geometries. This work demonstrates for the first time an alternative mechanism to realizing hollow core fibers based on ENZ/zero-index materials. It enriches the understanding of using ENZ material to enhance a fiber’s air-guiding capability, and can find applications in various processes, especially those that require high transmission speed, high intensity, or gas/chemical-filled fiber core.

## 4 Methods

### 4.1 Numerical simulation

Simulation of the nanostructures on the fiber was carried out using the MODE solver from Lumerical Solutions, Inc. The glass cladding was modeled using a ring of inner diameter *d*_*core*_. The outer diameter of the glass ring does not affect the result since the solver region was placed within the glass cladding to avoid using computation power to solve for non-core modes. The solver itself was set with perfectly matched layer (PML) [61] boundary condition, as well as symmetry condition on the −*x* boundary and anti-symmetry condition on the −*y* boundary, doing so will decrease the computation time while calculating the modes of interest. The minimum mesh size of the full solver region was set to be 200 nm, while an additional mesh override region with a mesh size of 20 nm was placed to cover the core region, since a finer mesh is required for any region with high amount of electric field to ensure the results’ accuracy.

In all simulations, permittivity function of silica is modeled using the Palik data [62]. For ITO, the complex frequency-dependent dielectric permittivity is described by Drude model with damping constant and plasma frequency obtained by ellipsometry measurement: *ε*_∞_ = 4.23, Γ = 2.41 × 10^14^ s^−1^, *ω*_p_ = 2.55 × 10^15^ s^−1^.

## Supporting information

Modelling of hollow core ENZ fiber with lower loss ENZ material; A note on the saturation of losses beyond 400 nm ENZ films; A note on the modes supported by ENZ thin films and high peak losses due to enhanced transmittance; Higher order guided mode of ENZ hollow core fiber; Group index and dispersion of the ENZ hollow core fiber’s core mode.

## Supplementary Material

Supplementary Material Details
