# Supplementary material for: Hollow core optical fiber enabled by epsilon-near-zero material
Source: Nanophotonics. 2024 Mar 6;13(7):1025–31. doi: 10.1515/nanoph-2024-0025 (PMC11501789; doi:10.1515/nanoph-2024-0025)
Supplement: Supplementary file 1 — Supplementary Material Details [file j_nanoph-2024-0025_suppl_001.docx]

**Supplementary materials:**

**Hollow Core Optical Fiber Enabled by Epsilon-Near-Zero Material**

*Leon Zhang^1,2^, Stuart Love^1,2^, Aleksei Anopchenko^1,2^, and Ho Wai Howard Lee^1,2*^*

^1^ Department of Physics & Astronomy, University of California, Irvine, CA 92697, United States

^2^ Beckman Laser Institute and Medical Clinic, University of California, Irvine, CA 92697, United States

*Corresponding author: Ho Wai Howard Lee ([Howardhw.lee@uci.edu](mailto:Howardhw.lee@uci.edu))

**Supplementary S1: Modelling of hollow core ENZ fiber with lower loss ENZ material.**

We simulated the fiber structure with a theoretical lower intrinsic loss material to explore its effect on the guiding efficiency of the hollow core optical fiber. Since intrinsic loss is directly related to the imaginary part of the permittivity (Im(ε)), we simulate a material with lower loss by modifying Im(ε) of an existing material, while leaving Re(ε), and subsequently, the ENZ wavelength, unchanged.


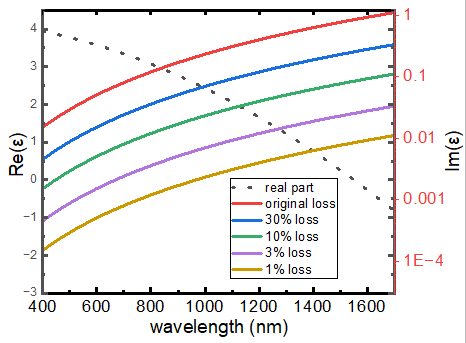


Fig. S1. Complex permittivity of the material used in simulation. The real part of permittivity was experimentally measured from an indium tin oxide sample and kept the same for all cases. The original loss is the experimentally measured loss of indium-doped tin oxide sample. The other losses were generated by taking a percentage of the original loss.

The Drude parameters of the base ITO material's permittivity can be found in the Methods section. The resulting dispersion data was used in the numeric solver to simulate the structure and calculate the fiber loss. We then simulated the theoretical lower loss ENZ materials by multiplying Im(ε) of the base material by a certain percentage; their dispersion is shown in Fig. S1. Finally, we can simulate the fiber structure with the replaced lower loss material (Fig. 4b in the main text).

**Supplementary S2: A note on the saturation of losses beyond 400nm ENZ films.**

Beyond 400 nm ENZ film thickness, the core mode losses saturate. In the thinner films we observe that there is an additional coupling to the ENZ thin film as shown in the figure below with the thinnest film 40 nm showing the highest level of confinement.


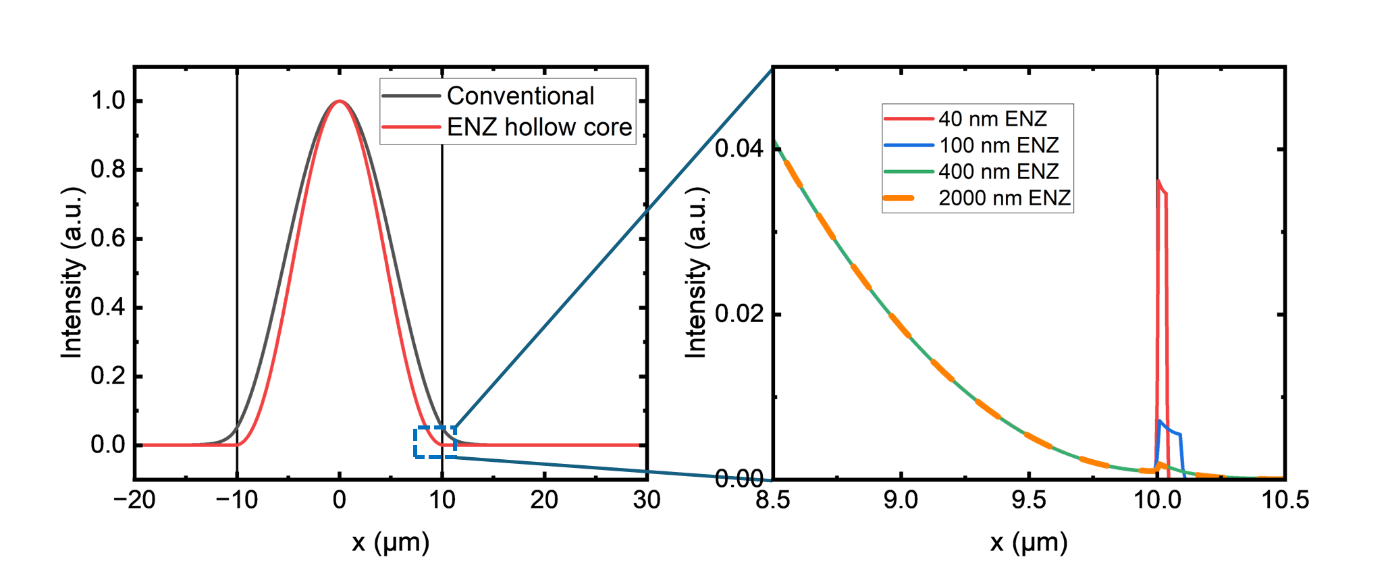


Fig. S2. A cross section of the field confinement of the ENZ film. The ENZ thin films of 40nm and 100 nm show confinement in the film.

In comparison with the main text in Fig. 3b, this above information supports the idea that the limited losses in the thinner films are due to this coupling to the ENZ modes. It is known that thin ENZ films can support strongly confined electric fields [6,7], and the ENZ mode excitation becomes weaker as thickness increases. Due to the lossy nature of ENZ modes, the fiber’s core mode will experience increased loss as it couples to the ENZ mode. For larger ENZ layer thickness, the layer can no longer support sufficiently strong electric field, and thus there are little interaction between fiber core mode and the ENZ mode, which leads to the saturation behavior for increasing ENZ layer thickness and fiber core mode attenuation as shown in Fig. 4 of the main text.

**Supplementary S3: A note on the modes supported by ENZ thin films and high peak losses due to enhanced transmittance.**

Phase matching conditions have been used to couple light from the core mode of single mode optical fiber to the ENZ mode of ENZ layer in our previous studies [1, 2]. This has been useful before when using silica core (traditional) fiber optics as the core mode can couple directly to the ENZ mode, enabling interaction. Though our main interest is reducing the loss of the core mode around the zero-cross wavelength of ENZ material, when phase matching exists between the ENZ layer and the cladding, large losses can take place.

While ITO thin films can naturally support various modes like lossy mode resonances [3] ENZ mode [4] and surface plasmon polariton mode [5] these modes remain unexcited in our case. This is because our fundamental mode travels through air, and such modes only get excited when light originates from a medium with a higher refractive index than the air outside.

In the simulated spectrum of our capillary fiber coated with ENZ, a sharp peak with pronounced losses occurs near the wavelength of 1300 nm (Fig. 4b in the main text). This effect is particularly evident in structures utilizing lower-loss ITO. To explore this phenomenon, we calculated the spectral dependence of transmittance through the ITO film on silica, varying the angle of incidence (Fig. S3). This calculation was performed using the transfer matrix method. Fig. S3 reveals a region of high transmittance, indicated by the white line. By extracting this white line, we determined the effective index associated with the enhanced transmittance:

$n_{eff}=n_{o}Sin(\theta)$.


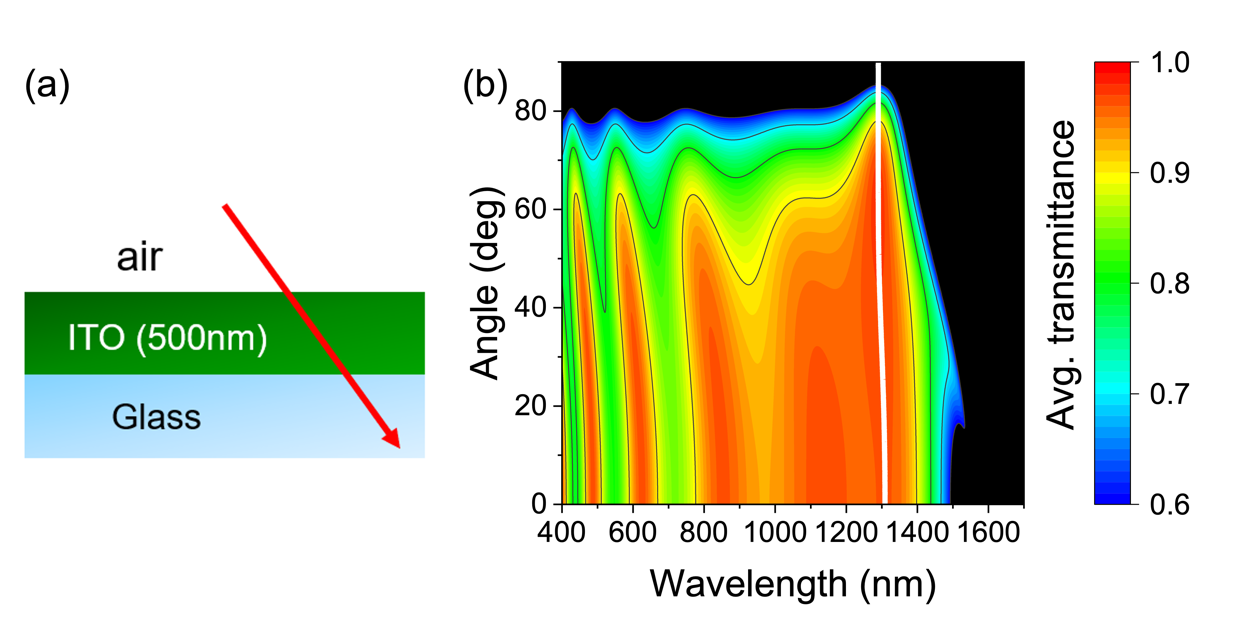


Fig. S3. (a) Schematic of planar geometry in the simulation. (b) The calculated transmittance of the planar ENZ film with a thickness of 500 nm on silica. The transmittance is averaged between *s*- and *p*-polarizations. The imaginary part of the ITO permittivity is 1% of the base permittivity. The high level of transmittance is observed at the wavelengths shown by the white line.

We then calculated the effective index dispersion and loss for the uncoated and coated silica capillary fiber with the ITO film of thickness 500 nm and ENZ crossing point of 1550 nm (with 1% loss) (see Fig. S4). The effective index dispersions and loss of the ENZ coated capillary fiber were calculated using Lumerical MODE optical simulator.

The dashed and solid red curves in Fig. S4 correspond to the effective index dispersions of the fundamental mode of the uncoated and coated capillary fibers, respectively. The strong loss peak at wavelength of 1300 nm in the simulated modal loss curve of the ENZ coated fiber (black solid curve) is due to phase matching between the fiber mode index and the index of the highly transmissive ENZ layer. At this wavelength light leaks from the core, leading to significant loss. Note that the calculations are shown for 500 nm thick 1% loss ITO only, and different ENZ material have additional phase matching condition at different wavelengths below the ENZ wavelength. The result shown in the Fig. 4b in the main text was calculated with a very large ENZ wall thickness of 50 µm to simplify interactions at the lower wavelength range, since the focus of the fiber structure is around the ENZ wavelength.


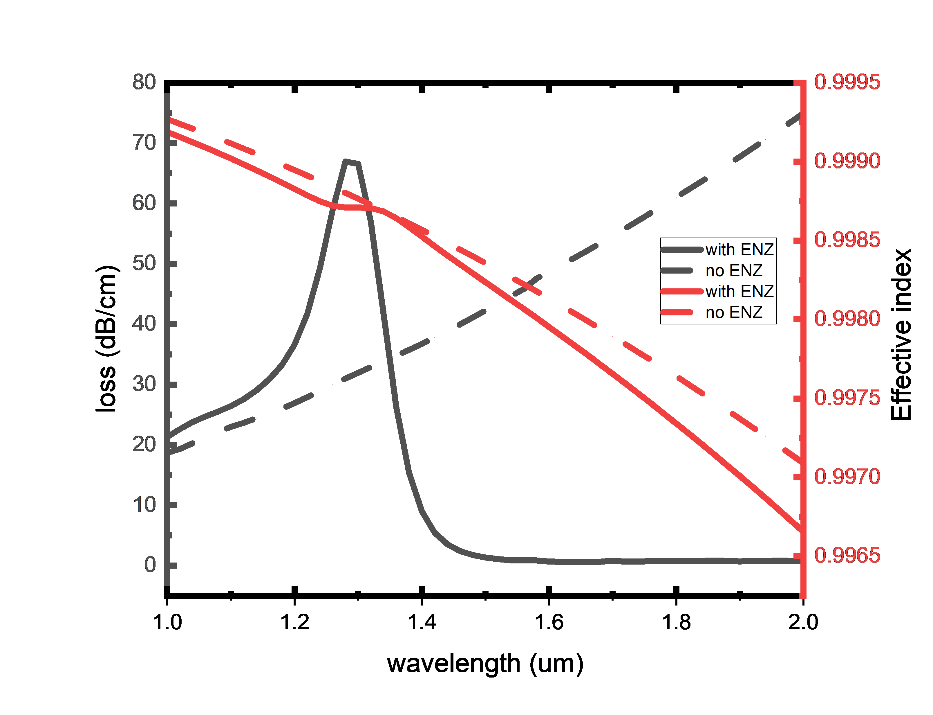


Fig. S4. Simulated ENZ coated and uncoated fiber’s effective index and loss (ENZ coated: solid curves, uncoated: dashed curves). Enhanced transmittance at the ENZ layer's refractive index, which coincides with the fiber mode index at 1300 nm, induces light leakage from the core and subsequent high loss.

**Supplementary S4: Higher order guided mode of ENZ hollow core fiber.**

Due to the high numerical aperture of the fiber design caused by the large index difference between the core and the cladding, the fiber supports many modes for all tested core diameters. At higher core diameters, the fiber is expected to support additional transverse modes, however, higher order modes generally have increased attenuation, and thus is not expected to influence the transmission over a significant distance, and therefore was not included in this work. Regarding the interaction between higher-order modes and the ENZ layer, we have investigated the modal intensity distribution of the first 15 higher-order modes for the 20 $\mu m$ core diameter case and have found that the normalized electric field intensity within the ENZ layer is always less than 10^-3^. Thus, it is seen that just like the fundamental mode, the higher-order modes have little interaction with the ENZ layer.

**Supplementary S5: Group index and dispersion of the ENZ hollow core fiber’s core mode.**

The group index was determined by performing modal calculations of the fundamental core mode of the ENZ fiber. Figure S5 illustrates the group index, with a noticeable dip at 1300 nm for the same reasons outlined earlier.


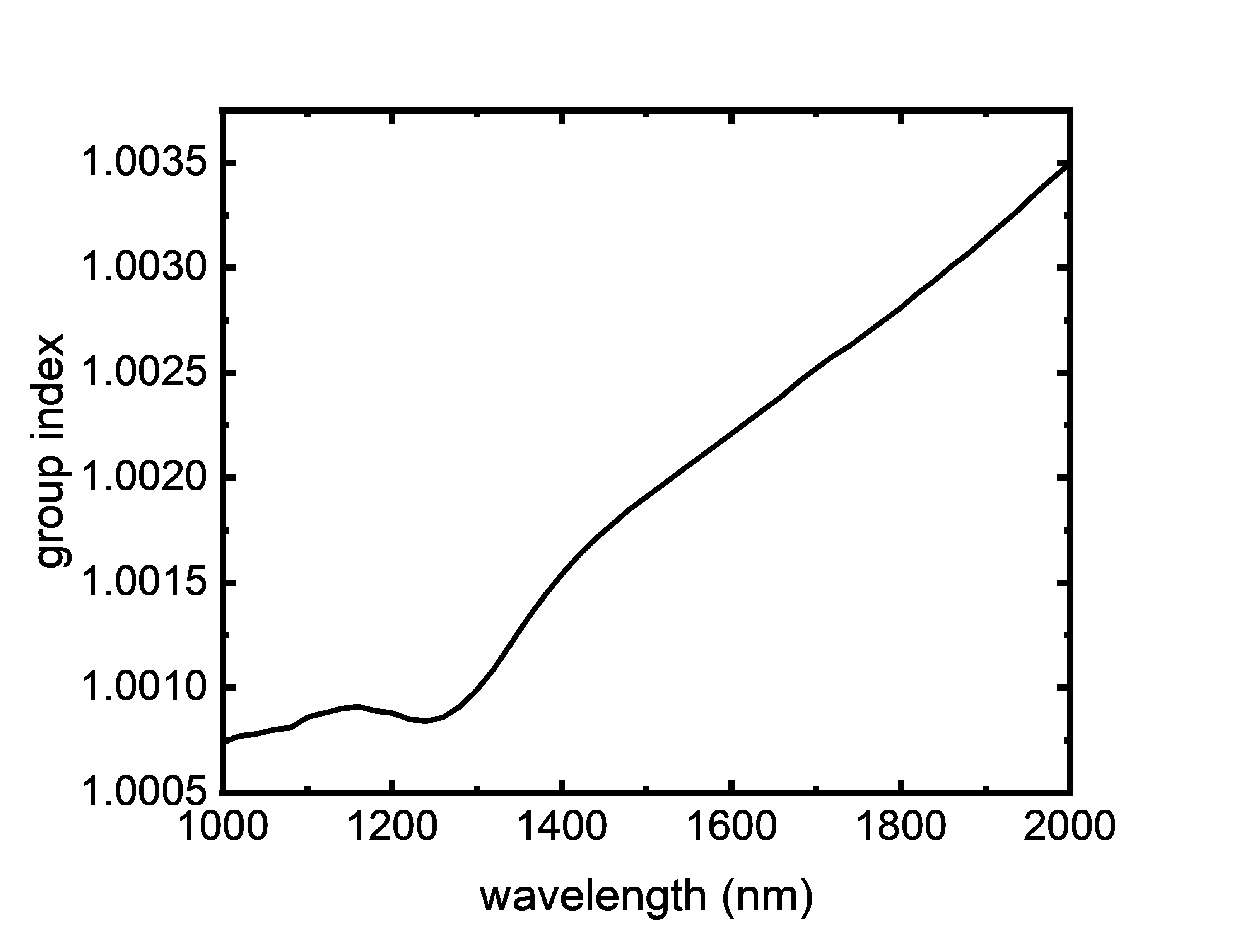


Fig. S5. Group index of the fundamental mode of the hollow core fiber. The core diameter is 20 **μm, and the ENZ layer thickness is 400 nm.**

References

[1] K. Minn, A. Anopchenko, J. Yang, and H. W. H. Lee, "Excitation of epsilon-near-zero resonance in ultra-thin indium tin oxide shell embedded nanostructured optical fiber," *Scientific Reports* 8, 2342 (2018)

[2] J. Yang, K. Minn, A. Anopchenko, S. Gurung, and H. W. H. Lee, "Excitation of Epsilon-Near-Zero Mode in Optical Fiber," *Laser & Photonics Reviews* 17, 2200539 (2023).

[3] I. D. Villar, C. R. Zamarreño, M. Hernaez, F. J. Arregui, and I. R. Matias, "Generation of Lossy Mode Resonances With Absorbing Thin-Films," *Journal of Lightwave Technology* 28, 3351-3357 (2010)

[4] S. Campione, I. Brener, and F. Marquier, "Theory of epsilon-near-zero modes in ultrathin films," *Physical Review B* 91, 121408 (2015).

[5] F. Michelotti, L. Dominici, E. Descrovi, N. Danz, and F. Menchini, "Thickness dependence of surface plasmon polariton dispersion in transparent conducting oxide films at 1.55 μm," *Optics Letters* 34, 839-841 (2009).

[6] O. Reshef, I. De Leon, M. Z. Alam, and R. W. Boyd, "Nonlinear Optical Effects in Epsilon-near-Zero Media", Nat Rev Mater **4**, 8 (2019).

[7] A. Anopchenko, S. Gurung, S. Bej, and H. W. Lee, “Field Enhancement of Epsilon-near-Zero Modes in Realistic Ultra-Thin Absorbing Films,” Nanophotonics 12, 2913 (2023)
